# Biomass-derived carbon quantum dots for the fabrication of a durable, self-cleaning, and corrosion-resistant superhydrophobic coating on steel

**DOI:** 10.1038/s41598-026-47261-8

**Published:** 2026-04-30

**Authors:** M. E. Mohamed, B. A. Abd-El-Nabey, A. Ezzat

**Affiliations:** 1 Chemistry Department, Faculty of Science, Alexandria University, Alexandria, Egypt; 2 Faculty of Advanced Basic Sciences, Alamein International University, New Alamein City, Matrouh Governorate Egypt

**Keywords:** Superhydrophobic coating, Carbon quantum dots (CQDs), Biomass-derived materials, Green synthesis, Corrosion resistance, Self-cleaning, Mechanical durability., Chemistry, Engineering, Environmental sciences, Materials science

## Abstract

The enhancement of durable and environmentally friendly superhydrophobic (SHP) coatings for metal protection remains a significant challenge. This study presents a green and effective strategy for fabricating a robust SHP coating on steel by integrating carbon quantum dots derived from Conocarpus lancifolius biomass (denoted C-CQDs). The pivotal innovation lies in the incorporation of C-CQDs during a one-step electrodeposition process, which fundamentally alters the coating’s growth mechanism to promote nucleation, resulting in a dense, finely granular morphology. This unique structure, confirmed by SEM and AFM, achieved a substantial surface roughness of 335.6 nm and exceptional water repellency, having a water contact angle of 167° and a water sliding angle of 1°. Compared with the C-CQD-free SHP coating, the C-CQD-containing coating showed improved abrasion tolerance under the present test conditions (maintaining superhydrophobicity after 900 mm of abrasion) and outstanding chemical stability across a wide pH range (1–13). Electrochemical tests revealed a remarkable corrosion protection efficiency of 93.1%, a significant increase from the 78.5% efficiency of the C-CQD-free SHP coating. This work not only introduces a sustainable source for high-performance nanomaterials but also provides a versatile strategy for designing next-generation functional coatings with integrated anti-corrosion, self-cleaning, and mechanical resilience for practical industrial applications.

**Supplementary Information:**

The online version contains supplementary material available at 10.1038/s41598-026-47261-8.

## Introduction

Surfaces that strongly repel water, often referred to as Superhydrophobic (SHP) surfaces, have drawn significant scientific interest over the past few decades due to their distinctive wetting behavior and the breadth of their technological applications^1^. In nature, such properties are exemplified by lotus leaves, rose petals, and the legs of water striders, where a combination of surface roughness having micro- and nanostructures with low surface energy leads to the minimization of water adhesion. This results in water droplets forming nearly spherical shapes and rolling off the surface with minimal resistance, carrying away contaminants in the process^2^. Quantitatively, SHP surfaces are typically defined by exhibiting a water contact angle (WCA) more than 150° and a water sliding angle (WSA) below 10°, parameters that collectively define their extreme non-wettability^3^.

Due to these characteristics, SHP surfaces were explored for various applications, for instance, self-cleaning systems^4^, biomedical devices^5^, anti-icing coatings^6^, antifouling materials^7^, corrosion protection^1^, oil-water separation^2^, and microfluidics^8^. Among these, corrosion resistance and self-cleaning stand out as particularly relevant to environmental sustainability. Steel, for example, is widely used in structural and marine environments but is highly susceptible to corrosion, leading to costly maintenance, structural failures, and safety hazards^9^. SHP coatings, due to their water-repellent nature, offer a promising solution to protect metals from corrosion while maintaining durability^10^ and self-cleaning functionality^4^. Self-cleaning has obtained significant consideration owing to its wide relevance in glass, solar panels, and construction materials, offering benefits such as reduced maintenance costs, less manual effort, and faster cleaning^11^.

The preparation of SHP surfaces typically requires methods that can both build micro/nanostructured roughness and introduce low-surface-energy chemistry^12^. Techniques such as chemical vapor deposition^13^, sol–gel processing^14^, plasma treatment^15^,, spray-coating^16^, and electrodeposition^17^ have been investigated. Electrodeposition is considered a particularly attractive route for fabricating superhydrophobic (SHP) coatings intended for corrosion protection because it combines industrial maturity with controllable microstructure formation. Compared with many alternative fabrication techniques that may require expensive equipment, complex processing steps, or environmentally problematic reagents, electrodeposition typically uses a simple apparatus (power supply, electrodes, and electrolyte bath) and offers low processing cost and high production efficiency. Importantly, the surface morphology and hierarchical roughness—key factors governing SHP behavior—can be readily tuned by adjusting deposition parameters such as voltage/current and deposition time, leading to good repeatability. In addition, electrodeposition is not restricted by substrate shape or size and can be applied to complex components, making it suitable for batch processing and scalable manufacturing^18^.

Despite substantial progress, several challenges still hinder the broader adoption of SHP coatings in practical applications. Many reported coatings rely on fluorinated compounds such as perfluorinated silanes or fluorocarbon molecules to achieve ultralow surface energies. Although highly effective, these chemicals are known for their environmental persistence, potential toxicity, and bioaccumulation in living organisms^19^. Furthermore, SHP coatings often suffer from poor mechanical durability, chemical instability, and limited resistance to abrasion, which restricts their long-term functionality in real-world conditions^20^. These limitations underscore the urgent need for environmentally benign, mechanically robust, and economically viable SHP coatings.

In practice, the main difficulty in constructing long-lasting SHP coatings is that the micro/nanostructures responsible for the Cassie–Baxter state are mechanically fragile. Abrasion, particle impact, and handling can fracture or flatten surface asperities, reducing hierarchical roughness and promoting a Cassie-to-Wenzel transition, which increases adhesion and degrades water repellency^21^. Chemical instability is also common because many SHP coatings rely on low-surface-energy modifiers that can hydrolyze, oxidize, or detach under strong acid/alkali or saline environments^22^. In addition, pores and microdefects created during coating formation can act as fast transport pathways for electrolytes, enabling underfilm corrosion^18^. Therefore, simultaneously improving coating compactness, structural robustness, and interfacial bonding—while maintaining fluorine-free chemistry—remains a key challenge for practical SHP metal protection.

In recent times, carbon-based nanomaterials have been investigated as promising components to enhance the performance of SHP surfaces^23^. Carbon quantum dots (CQDs), a relatively novel member of the carbon nanomaterial family, possess unique properties including nanometer-scale dimensions, tunable surface chemistry, and abundant functional groups. These attributes make them highly effective in increasing surface roughness and promoting strong interactions with low-surface-energy modifiers^24^. Moreover, CQDs can be synthesized from renewable biomass resources, offering an eco-friendly and low-cost alternative to conventional synthesis routes that often require hazardous chemicals or energy-intensive processes. Biomass-derived CQDs not only reduce synthesis-related environmental impacts but also contribute to waste valorization, turning agricultural by-products into high-value functional nanomaterials^25^.

In our previous work, carbon-based nanomaterials were successfully employed to construct SHP coatings. For example, graphene-modified electrodeposition on steel followed by fatty acid treatment produced surfaces with WCA of ~ 161.4°, WSA of 1°, and superior corrosion and abrasion resistance, highlighting the role of carbon-derived nanostructures in enhancing SHP performance on metallic substrates^1^. Also, green-synthesized CQDs obtained from banana peel biomass achieved WCA of ~ 163°, near-zero WSA (~ 1°), and high durability under harsh pH and abrasion conditions^2^. A sol–gel CQD-based coating significantly reduced corrosion current density exceeding two orders of magnitude while sustaining a WCA of ~ 158°, confirming the protective potential of CQDs on steel substrates^26^. Recently, several types of CQDs derived from biowaste have been developed through eco-friendly synthesis routes and characterized using various surface analysis techniques. For example, lupine-based CQDs (LCQDs) were synthesized via a hydrothermal process and studied by multiple spectroscopic and microscopic methods^27^. Similarly, damsissa CQDs (DCQDs) were incorporated into permanganate phosphate conversion coatings on steel substrates, where surface investigations including XRD, TEM, EDX, and XPS confirmed their uniform distribution and strong adhesion on the steel surface^28^. Moreover, fenugreek-derived CQDs (FCQDs) were prepared using a one-step pyrolysis approach and subjected to comprehensive characterization, such as SEM and XRD, to examine their structural and morphological properties^29^. In addition, ginger waste has recently been used as a sustainable source for producing CQDs, where the obtained ginger-based CQDs (GCQDs) exhibited excellent optical features and potential applications in bioimaging and sensing, further emphasizing the versatility of biowaste-derived resources in CQDs fabrication^25^.

Conocarpus lancifolius, belonging to the Combretaceae family, is a fast-growing evergreen adaptable to a range of sizes, often growing with multiple trunks in a shrub-like habit but capable of reaching heights of up to 20 m. Native to East Africa and the Arabian Peninsula, it is known locally by names such as “Damas” in Arabic and “Qalab” in Somali. Due to its exceptional tolerance to high salinity and drought, it is considered a pioneering species for reforestation in arid environments and has been widely adopted for landscaping in residential areas and along roadsides throughout the Gulf region. This extensive cultivation and rapid growth generate a substantial amount of biomass from leaf litter and trimmings, which is often discarded as waste^30^. Given its high lignocellulosic composition, this abundant, low-cost, and renewable biomass makes Conocarpus lancifolius an excellent and sustainable precursor for synthesizing carbon-based nanomaterials, such as CQDs used in this study.

In this study, we demonstrate a sustainable approach to construct SHP coatings on steel substrates. A hierarchical micro-nanoscale roughness, the primary criterion for superhydrophobicity, was engineered through an electrodeposition process. To augment the nanoscale texture, we utilized CQDs synthesized from Conocarpus lancifolius biomass (C-CQDs). The second criterion was met by chemically treating the roughened surface with a biodegradable low-surface-energy substance, stearic acid, to achieve the desired non-wetting state. The composition and morphology of the prepared CQDs were analyzed using FTIR, XRD, TEM, EDX, and XPS. The prepared SHP coatings were thoroughly characterized for surface morphology (SEM, AFM), composition (EDX, XPS), and wettability (WCA, WSA). Their mechanical robustness was evaluated through abrasion tests on sandpaper, while chemical stability was assessed by exposure to solutions having different pH (1 to 13). Corrosion resistance was determined using electrochemical impedance spectroscopy (EIS) and potentiodynamic polarization tests in 0.5 M NaCl solution. The innovation of this work stems from the synergistic use of biomass-derived C-CQDs synthesized from *Conocarpus lancifolius* as nanoscale roughness enhancers within an eco-friendly electrodeposition process to fabricate superhydrophobic coatings on steel. By promoting a dense hierarchical texture and limiting defects that facilitate electrolyte transport, the C-CQDs-assisted coating provides a robust platform that combines water repellency with improved mechanical/chemical stability and corrosion resistance. This strategy highlights a scalable and sustainable pathway to replace conventional synthetic nanofillers with naturally sourced carbon nanomaterials without sacrificing protective performance.

## Materials and methods

### Materials

The used chemicals were of analytical grade and included stearic acid (SA), anhydrous ethanol, sulfuric acid, sodium chloride, nickel chloride hexahydrate, boric acid, nickel sulfate, and sodium hydroxide, which were supplied by Chematek Company (Egypt). Conocarpus lancifolius biomass was procured from a local marketplace. The experiments were performed on steel substrates with dimensions of 50 mm × 20 mm × 1.0 mm. To confine the electrodeposition to a specific region, the substrates were masked with an epoxy resin, exposing a defined geometric area of 20 mm × 10 mm for the coating process.

### Preparation methods

#### Synthesis of Conocarpus lancifolius quantum dots (C-CQDs)

Fresh leaves of Conocarpus lancifolius (3 g) were thoroughly cleaned with distilled water, air-dried, and finely ground. The resulting powder was dispersed in 30 mL of distilled water with 15 min of stirring at ambient temperature. The obtained suspension was introduced into a Teflon-lined autoclave and subjected to hydrothermal treatment at 200 °C for 3 h. After naturally cooling under ambient conditions, the carbon-rich solution was diluted with 50 mL double distilled water and centrifuged at 10,000 rpm for 15 min to eliminate larger particulates. The supernatant was afterward delivered through a 0.22 μm syringe filter, producing a pale-brown C-CQDs solution. The concentration of the C-CQDs was determined in ppm^2^.

#### SHP coating preparation on steel substrate

Steel plates were first prepared by sequential polishing with emery papers of increasing fineness, beginning with grade 300 and ending with grade 1000. After polishing, the substrates were cleaned in a soap (detergent) solution for 10 min, briefly activated in 2.0 M sulfuric acid for 30 s, and thoroughly rinsed with distilled water and ethanol. Electrodeposition of nickel and nickel/carbon quantum dot (Ni–C-CQDs) coatings was then carried out under the conditions listed in Table 1. A platinum sheet with the same dimensions as the working electrode was used as the anode and positioned 20 mm from the cathode (steel substrate). After deposition, the coatings were rinsed with distilled water and dried under ambient conditions for 24 h. The dried samples were subsequently immersed in a 0.01 M ethanolic solution of stearic acid (SA) for 15 min and then dried again under ambient conditions. Various characterization and performance tests were performed on the Ni coating modified with stearic acid (Ni–SA) and the Ni–C-CQDs coating modified with stearic acid (Ni–C-CQDs–SA)^1^.

**Table 1:** Operating settings and bath compositions for the electrodeposition of Ni and Ni-CQD coating on the working electrode (steel).

Parameter		Level	
		Ni	Ni- CQDs
(Source of nickel ion)	NiCl_2_.6H_2_O	40 gL^− 1^	
	NiSO_4_	176 gL^− 1^	
(Buffer)	H_3_BO_3_	60 gL^− 1^	
The used deposition		6.0 min	
Deposition potential		11.0 V	
C-CQDs		0.0 gL^− 1^	0.0125, 0.025, and 0.05 gL^− 1^

### Characterization of the prepared C-CQDs

Fourier-transform infrared spectroscopy (FTIR, Bruker Tensor 37) was employed to identify the functional groups and chemical composition of the synthesized CQDs. The bonding states and elemental composition of C-CQDs were further inspected by X-ray photoelectron spectroscopy (XPS, Thermo Fisher Scientific, USA) via monochromatic Al Kα radiation, with a spot size of 400 μm, operating pressure of 10^−9^ mbar, and pass energies of 200 eV for survey scans and 50 eV for high-resolution spectra. Crystalline characteristics were investigated through X-ray diffraction (XRD, Bruker D2 Phaser) using Cu Kα radiation (λ = 0.154056 nm). The elemental distribution of the sample was assessed via energy-dispersive X-ray spectroscopy (EDX, JEM-2100, JEOL, Japan). Finally, the morphology and particle size of the CQDs were depicted by transmission electron microscopy (TEM, JEOL 1400 Plus, Tokyo, Japan).

### Characterization of the prepared SHP coatings

The morphology of the produced SHP coatings was inspected using a scanning electron microscope (SEM; JEOL, model JSM-200 IT). An energy dispersive X-ray spectrometer (EDX JEM-2100 Japan) was utilized to evaluate the composition of the formed SHP coatings. An optical contact angle goniometer (model 190-F2, Rame-hart CA instrument) was utilized to estimate the WCA and WSA with 5 µL deionized water droplets. For each sample, measurements were taken at different positions; values are reported as mean ± standard deviation (SD). The surface topography was characterized via atomic force microscopy (AFM) using a Shimadzu SPM9600 instrument. XPS examination was conducted to inspect the surface composition and chemical states of the SHP coatings on carbon steel with and without C-CQDs.

### Mechanical abrasion, chemical stability, and corrosion resistance of the prepared SHP coated steel

The mechanical abrasion characteristics of the generated SHP coats were assessed using a linear sandpaper abrasion test. The coated samples were placed coating-side down on 800-mesh sandpaper and subjected to a normal pressure of 3.0 kPa. The WSA and WCA were recorded after every 100 mm of abrasion distance after the prepared SHP sample was shifted horizontally. The described mechanical abrasion resistance represents the average of measurements obtained from two independently prepared samples.

To evaluate the chemical stability, the prepared SHP coatings were submerged in beakers with aqueous solutions of varying pH values (1–13). The pH was adjusted via sodium hydroxide and sulfuric acid. For each pH condition, WCAs and SAs were measured, and the reported values represent the average of tests on two independently prepared samples.

A frequency response analyzer potentiostat (PARSTAT, USA) was used to perform the electrochemical experiments in a three-electrode cell using an aqueous solution of 0.5 M NaCl at ambient conditions. The reference electrode was an Ag/AgCl electrode, and the counter electrode was a graphite rod. Working electrodes were made of bare steel and steel covered with SHP Ni-SA and Ni-CQDs-SA coats. Prior to electrochemical examinations to reach the equilibrium potential, the working electrode was put in a cell with a solution of 0.5 M NaCl that had been opened to the atmosphere at ambient temperature and left for 20 min. Electrochemical impedance spectroscopy (EIS) was performed with a 10 mV amplitude signal applied at the open-circuit potential and a frequency range of 0.01 ≤ f ≤ 1.0 × 10^6^. The potentiodynamic polarization measurements were carried out with a potential range of ± 250 mV around the equilibrium potential and at a scan rate of 0.5 mV/Second. The corrosion products of the SHP coated steel in the absence and presence of the optimum concentration of the C-CQDs after immersion in 0.5 M NaCl for 24 h was investigated via EDX technique. Measurement accuracy was confirmed by double-checking the experiments, and the results were within a 2% error range.

### Self-cleaning performance test

The performance of the self-cleaning of the SHP coating was evaluated by dispersing a uniform layer of dust onto the inclined surface. The water droplets rolling across the surface were then observed to effectively remove the contaminant particles. A video of this process is provided in supplementary information.

## Findings and discussion

### Characterization of the CQD

The prepared C-CQDs were thoroughly investigated by multiple analytical techniques to evaluate their morphology, composition, particle size, and surface chemistry. A combination of characterization methods, including FTIR, TEM, XRD, XPS, and EDX, was employed to provide a comprehensive understanding of their structural and chemical features.

**Fig. 1:**
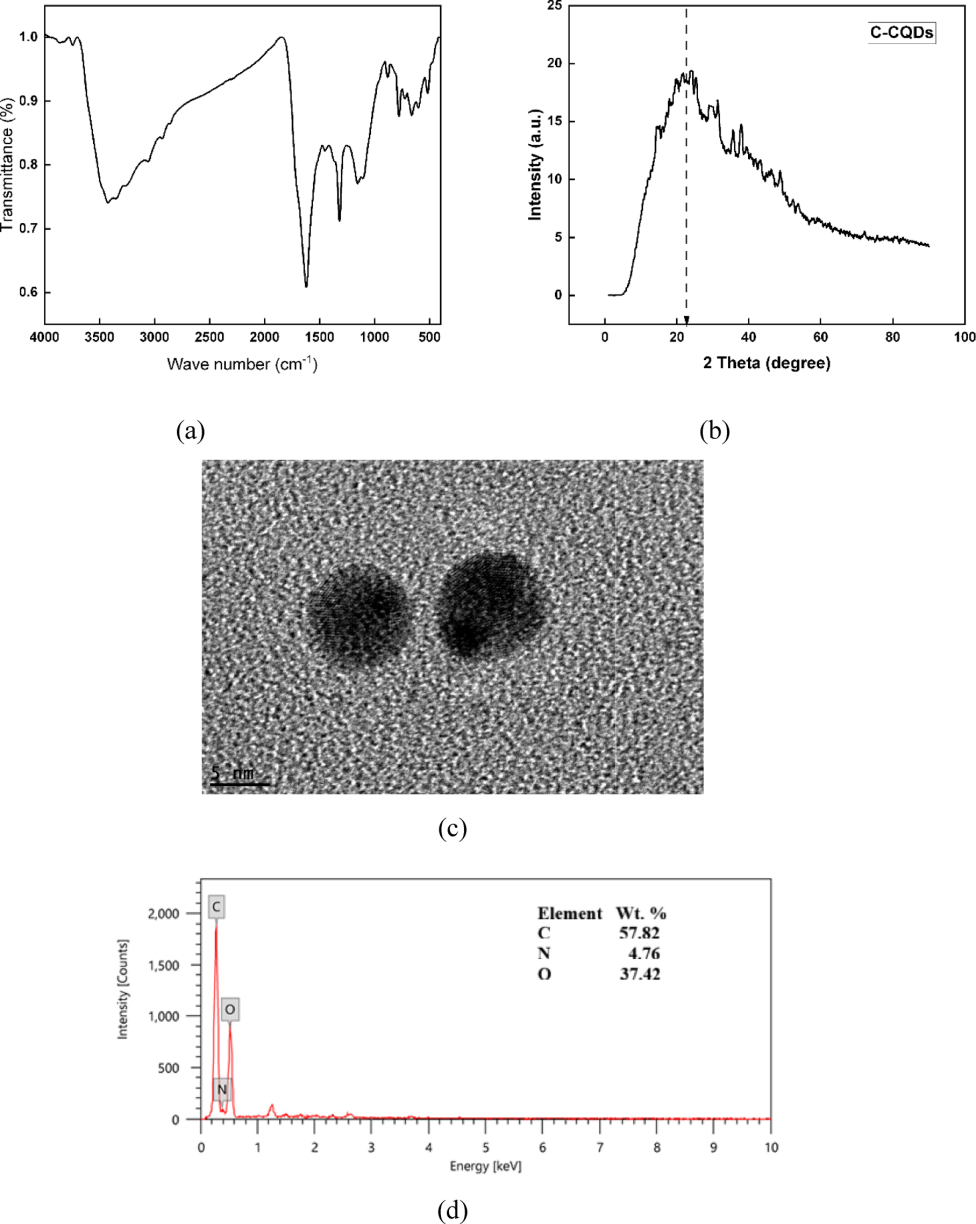
Characterization findings of C-CQDs: (**a**) FTIR, (**b**) XRD, (**c**) TEM, and (**d**) EDX.

The FTIR spectrum of the produced C-CQDs (Fig. 1a) confirms the presence of several functional groups on their surface. The broad absorption band around 3400 cm^−1^ is due to the stretching vibrations of O–H/N–H, demonstrating hydroxyl and amine groups, which improve the hydrophilicity and dispersibility of CQDs in water. The band near 2920–2850 cm^−1^ is attributed to the stretching vibrations of aliphatic C–H groups. The strong absorption peak appeared at ~ 1630 cm^−1^ is attributed to C = O stretching of carbonyl groups or C = C stretching from aromatic domains. The peaks in the region 1380–1450 cm^−1^ are related to the stretching vibrations of C–N, while the bands between 1000 and 1200 cm^−1^ are correlated with C–O–C and C–O stretching modes. The characteristic peaks below 800 cm^−1^ are attributed to the bending vibrations of aromatic C–H bonds. These results confirm that the CQDs are rich in nitrogen- and oxygen-containing functional groups, which can facilitate further surface modification and broaden their potential applications^31^.

The XRD pattern of the synthesized C-CQDs (Fig. 1b) displays a broad diffraction peak centered around 2θ ≈ 22.7°, which is typically associated with the (002) lattice plane of graphitic carbon^32^. The broad and weak profile of this reflection indicates the small size and amorphous structure of the CQDs, suggesting a low degree of crystallinity and the presence of disordered carbon domains^33^. Such a pattern is consistent with the abundance of oxygen-containing functional groups on the surface, which disrupts the long-range graphitic order. These findings confirm that the prepared C-CQDs possess a predominantly amorphous carbon framework with partially graphitized domains—a structural feature that supports their optical activity and surface functionality.

The TEM micrograph (Fig. 1c) reveals that the Conocarpus-derived C-CQDs are nearly spherical and well-dispersed without noticeable aggregation, confirming their uniform nanostructure. The mean particle size was determined to be around 5 nm, indicating the successful formation of quantum-sized carbon nanoparticles. Such ultra-small dimensions are beneficial for enhancing the surface activity and interaction of CQDs with the substrate during the coating process. The nanoscale size also contributes to increased surface roughness and improved dispersion within the matrix, thereby facilitating the development of a more compact and uniform SHP coating on the steel surface^34^.

The EDX analysis (Fig. 1d) was conducted to assess the elemental composition of the prepared C-CQDs. The results imply that the nanoparticles are predominantly composed of carbon (57.74%), oxygen (37.46%), and nitrogen (4.62%).

**Fig. 2:**
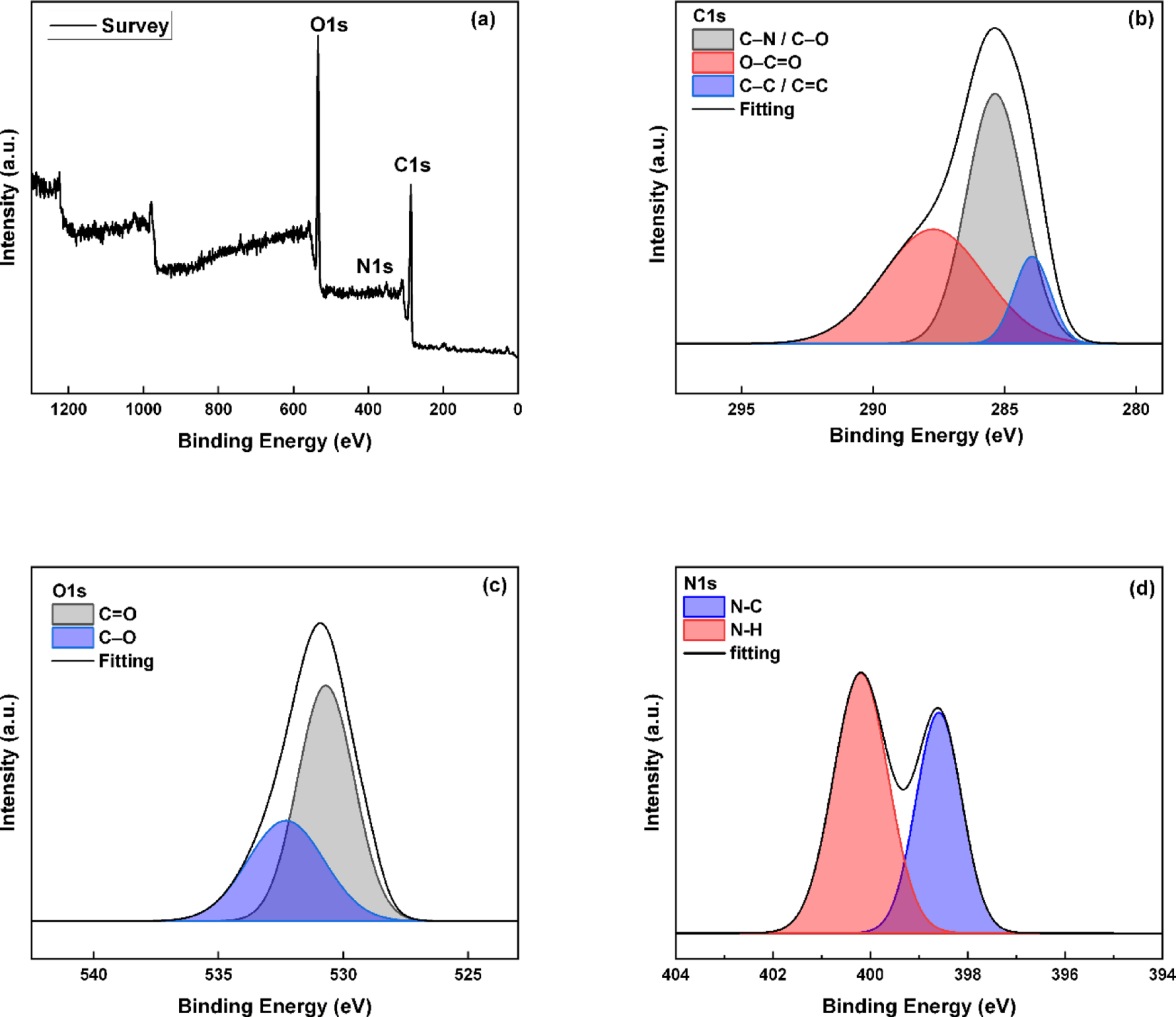
High-resolution XPS spectra of C-CQDs: (**a**) full survey; (**b**) C1s; (**c**) O1s; (**d**) N1s.

To further examine the surface bonding states and chemical composition of the synthesized C-CQDs, an XPS test was conducted. The XPS survey spectrum (Fig. 2a) confirms the presence of the main elements C, O, and N, consistent with the EDX results^31,35^. The high-resolution C 1 s spectrum (Fig. 2b) can be divided into numerous characteristic peaks corresponding to different carbon bonding environments. The main peak at 284.6 eV is due to C–C/C = C bonds in the sp²-hybridized carbon network, while peaks at 287.8 eV and 285.6 eV correspond to O–C = O and C–O/C–N functional groups^31,35^, respectively, indicating the coexistence of graphitic carbon and oxygenated carbon species.

The O1s spectrum (Fig. 2c) shows two main components located at 530.8 eV and 532.5 eV, which are due to C = O and C–O/C–OH bonds^36^, respectively, indicating the abundance of oxygen-containing functional groups. The N 1 s spectrum (Fig. 2d) exhibits two peaks at 400.2 eV and 398.5 eV, which can be attributed to pyrrolic and graphitic nitrogen species^37^. The presence of nitrogen atoms incorporated into the carbon lattice suggests successful heteroatom doping during the synthesis process.

These data confirm that the C-CQDs possess a surface enriched with nitrogen- and oxygen-containing functional groups, which enhance their hydrophilicity, chemical activity, and potential interaction with metal substrates. Such chemical features serve a crucial role in improving the dispersion and adhesion of the nanoparticles within the coating matrix, contributing to enhanced corrosion resistance and SHP performance.

### Characterization of the SHP coating on steel

#### EDX and SEM findings

**Fig. 3:**
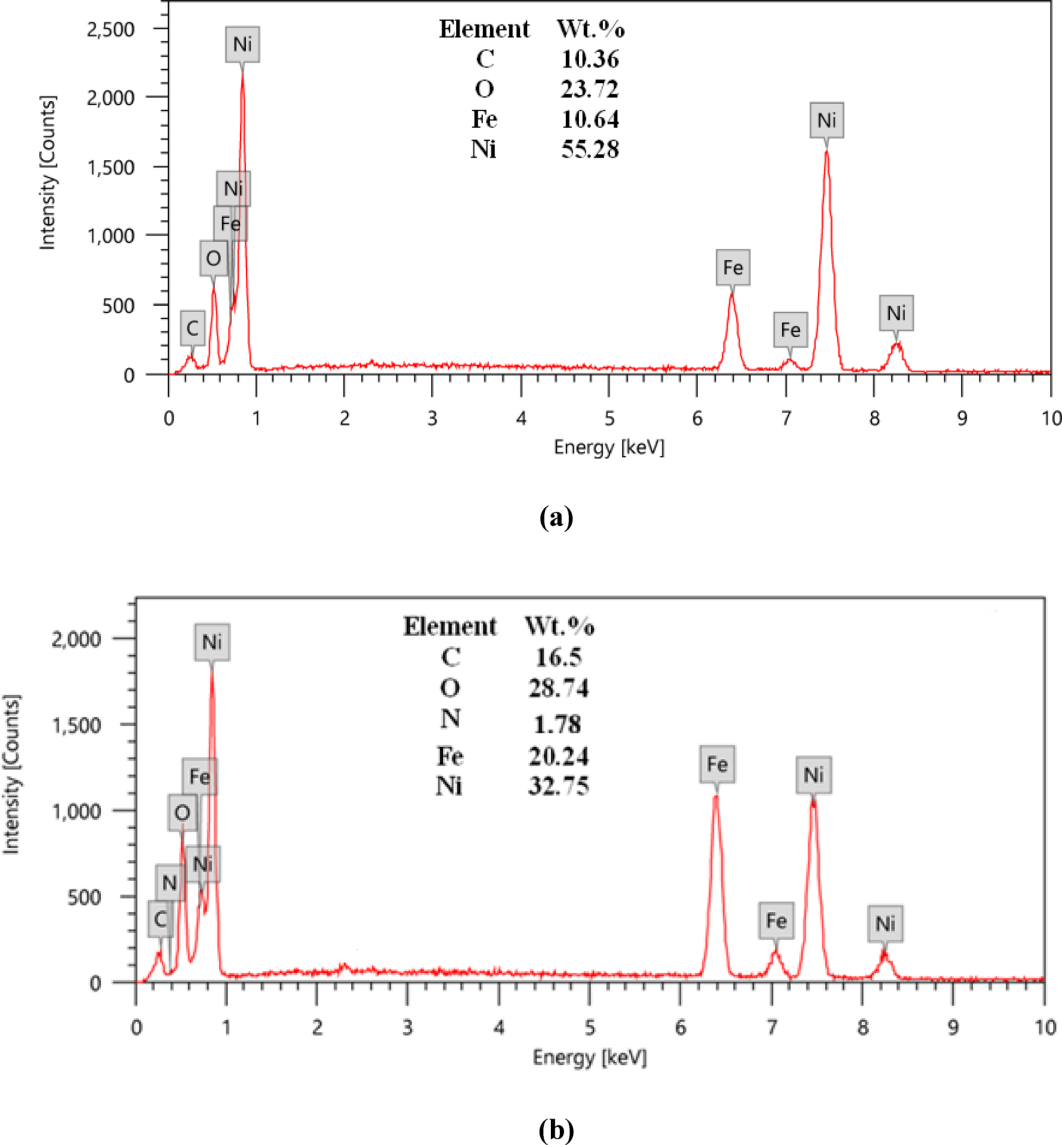
EDX spectra of: (**a**) SHP coating on steel without C-CQDs, (**b**) SHP coating on steel incorporating 50 ppm C-CQDs.

The elemental composition of the SHP coatings was analyzed using EDX, as shown in Fig. 3. The EDX spectroscopy data delivers compelling evidence for the effective incorporation of C-CQDs within the SHP coating. The EDX spectrum of the baseline SHP coating without C-CQDs (Fig. 3a) confirms the expected elemental composition, primarily featuring nickel (Ni) from the electrodeposited layer, along with oxygen (O) and carbon (C). The presence of oxygen and carbon in this baseline sample can be attributed to the stearic acid modification, which is a long-chain aliphatic compound. The detection of an iron (Fe) signal is a key indicator of a relatively thin coating, as the electron beam can penetrate the film to excite the underlying steel substrate.

The critical finding is observed in the spectrum of the coating incorporating C-CQDs (Fig. 3b). Two significant changes are evident:

1. *Substantial Increase in Carbon and Oxygen Content*: The noticeable rise in the atomic percentages of carbon (C) and oxygen (O) directly signals the presence of the C-CQDs on the surface. Since C-CQDs are carbonaceous nanomaterials rich in oxygen-containing functional groups (e.g., carboxyl, hydroxyl) from their biomass precursor and synthesis, their incorporation naturally increases the overall concentration of these two elements in the analyzed region.
2. *Appearance of Nitrogen (N)*: This is a particularly strong indicator. The baseline coating (without C-CQDs) shows no nitrogen, as neither the nickel electrodeposit nor stearic acid contains this element. The unambiguous appearance of a nitrogen peak in Fig. 3b can be conclusively traced back to the C-CQDs. Therefore, nitrogen acts as a unique elemental fingerprint, confirming that the C-CQDs are not merely present but are integrated into the coating’s surface chemistry.

**Fig. 4:**
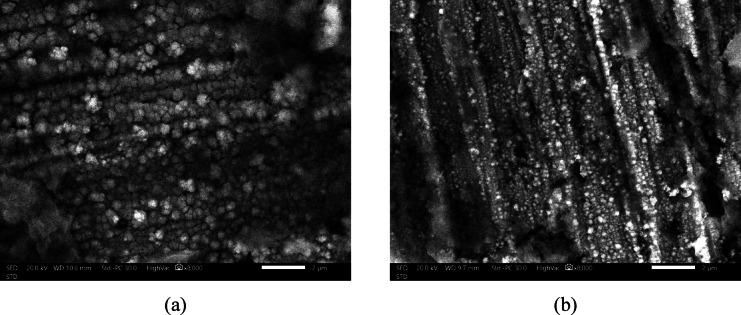
SEM spectra of: (**a**) SHP coating on steel without C-CQDs, (**b**) SHP coating on steel incorporating C-CQDs.

The observed EDX results, a decrease in Ni and an increase in Fe signal upon CQD incorporation, provide compelling evidence for a CQD-mediated alteration of the electrodeposition mechanism, which directly explains the enhanced superhydrophobicity. We propose that the CQDs act as numerous, highly active nucleation sites on the steel substrate. This significantly increases the rate of nucleation over the rate of crystal growth. Instead of forming a dense, continuous nickel film, this rapid nucleation leads to the formation of a larger number of smaller, finer grains. The outcome is a coating with a more granular, porous, and complex microstructure. This altered morphology has two direct consequences:

The SEM images, Fig. 4, provide direct visual evidence of the profound morphological changes induced by the incorporation of 50 ppm C-CQDs into the SHP coating, corroborating the mechanisms proposed from the EDX data.

Figure 4a, representing the SHP coating without C-CQDs, shows a surface composed of large and less dense micro-nano structures of a circular shape. This morphology is characteristic of a standard electrodeposition process where nickel crystallization proceeds with relatively few nucleation sites, allowing crystals to grow large and sparsely distributed. While this structure provides a degree of microscale roughness, its low density and large feature size limit the potential for creating the hierarchical micro-nanoscale roughness essential for robust superhydrophobicity.

In stark contrast, Fig. 4b reveals a dramatic transformation upon the incorporation of C-CQDs. The surface is now covered with significantly smaller, more densely packed circular structures. This distinct morphological shift is a direct consequence of the role of C-CQDs during electrodeposition. As previously hypothesized, the CQDs act as a massive number of dispersed nucleation centers. This drastically increases the nucleation rate over the crystal growth rate. Instead of a few crystals growing large, a multitude of smaller crystals nucleate simultaneously, leading to the observed fine-grained, dense, and uniform surface texture. The link between this observed morphology and the SHP performance is fundamental. The dense, hierarchical architecture of the C-CQD-incorporated coating is ideal for trapping air and minimizing liquid-solid contact. The smaller, more numerous features create a more complex surface topography, which, when chemically modified with the low-surface-energy stearic acid, results in a much more effective SHP state. This explains the superior water repellency, lower contact angle hysteresis, and enhanced self-cleaning performance observed for this coating compared to the baseline.

#### XPS results

**Fig. 5:**
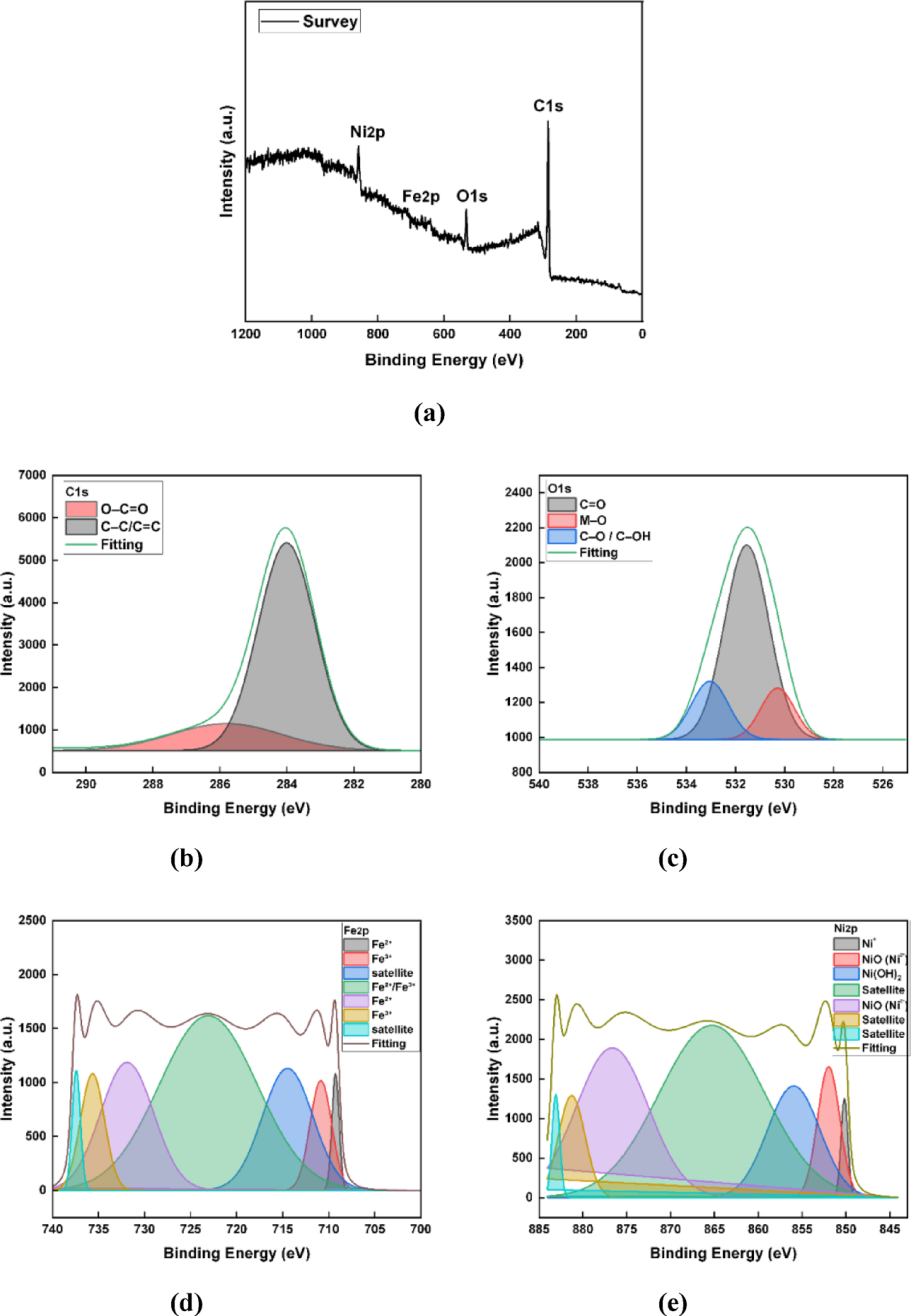
The XPS spectra of the SHP coatings on steel without C-CQDs: (**a**) full survey; (**b**) C1s; (**c**) O1s; (**d**) Fe2p; (**e**) Ni2p.

**Fig. 6:**
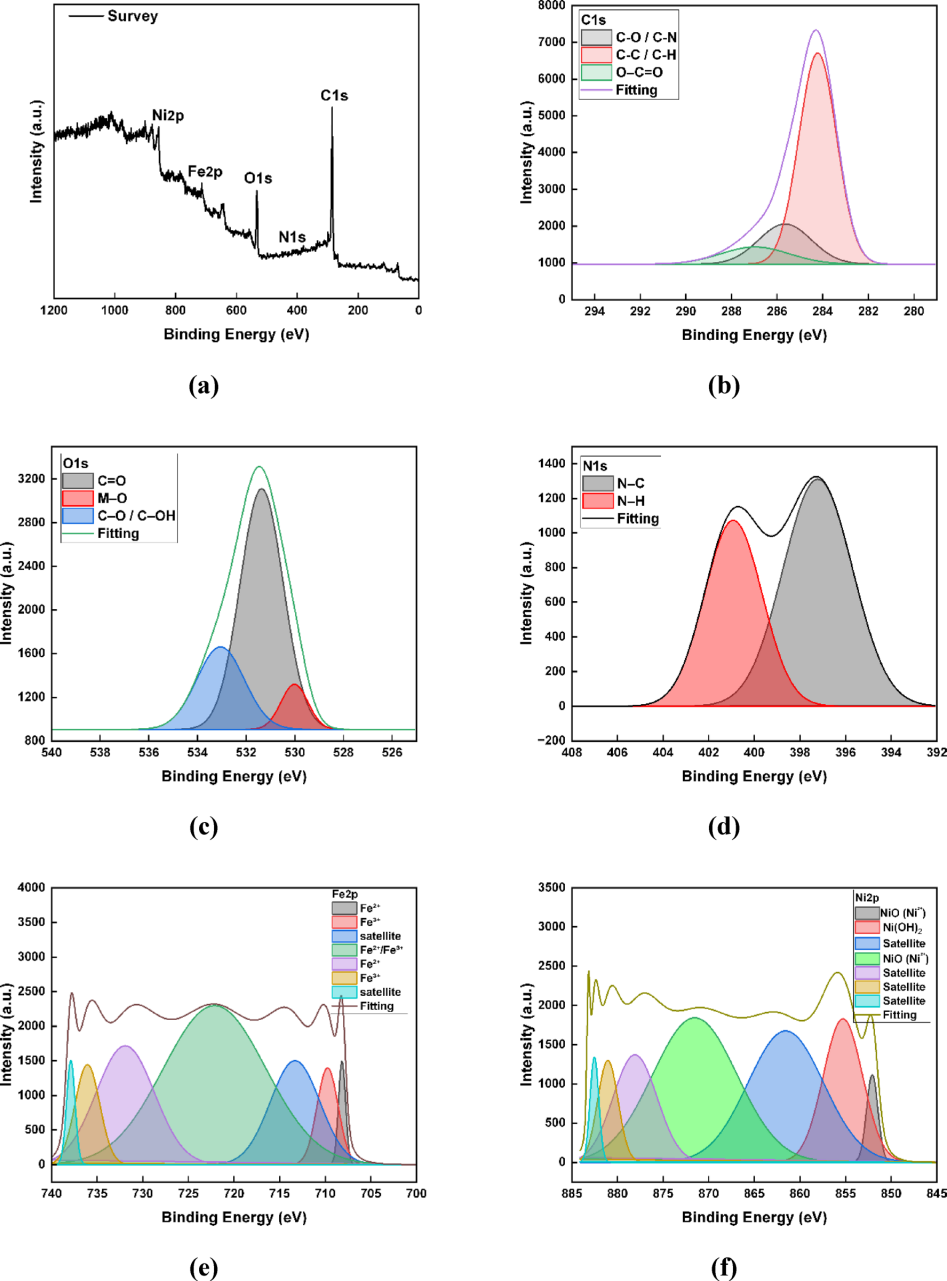
The XPS spectra of the SHP coatings on carbon steel with C-CQDs: (**a**) full survey; (**b**) C1s; (**c**) O1s; (**d**) N1s (**e**) Fe2p; (**f**) Ni2p.54.

The XPS analysis was operated to examine the chemical states and surface composition of the SHP coatings on steel with and without C-CQDs. The survey spectra (Figs. 5a and 6a) revealed the presence of C, O, Fe, and Ni in both coatings, while N was detected only in the coating containing C-CQDs, verifying the successful incorporation of nitrogen-rich groups from the quantum dots.

In the C 1 s spectrum, the CQD-based coating exhibited three peaks at 285.7, 284.2, and 287.3 eV (Fig. 6b), due to C–O/C–N, C–C/C = C, and C = O/O–C = O bonds^31,35^, respectively, indicating the presence of various oxygen- and nitrogen-containing functional groups introduced by CQDs. In contrast, the coating without CQDs (Fig. 5b) showed only two peaks (284.0 and 285.9 eV), reflecting a lower degree of surface functionalization. The N 1 s signal, appearing only in the CQD-modified coating (Fig. 6d), displayed peaks at 397.2 and 401 eV, attributed to pyridinic-N and graphitic/oxidized N species^37^, further confirming the nitrogen from C-CQDs. The O1s spectra (Figs. 5c and 6c) for both coatings contained peaks around 530, 531.5, and 533 eV, corresponding to metal–O, C = O/metal–O, and hydroxyl species^36^, respectively; however, the O 1 s intensity was significantly higher in the presence of CQDs, suggesting enhanced surface oxidation and stronger metal–oxygen–carbon interactions.

As further supported by the Fe 2p spectra (Figs. 5d and 6e), multiple overlapping peaks were observed, confirming the coexistence of several iron oxidation states and compounds within the coating layer. The peaks detected at approximately 709–710.8 eV correspond to Fe^2+^ species such as FeO or Fe_3_O_4_, while those at 713–714 eV are attributed to Fe^3+^ species, mainly Fe_2_O_3_ or FeOOH. The higher binding energy peaks around 723–724 eV and 735–737 eV are characteristic of Fe 2p_1/2_ components and their associated satellite peaks, respectively, reflecting partial oxidation and complex chemical environments on the coated surface^38^. Notably, the Fe 2p intensity was higher in the CQD-modified film, indicating a thinner yet more compact coating that partially exposes the underlying substrate. This enhancement suggests that the incorporation of CQDs improves film formation by promoting uniform nucleation and controlling crystal growth. Acting as active nucleation centers, CQDs accelerate the nucleation rate while suppressing crystal growth, leading to the formation of finer nanoscale structures. Consequently, the surface roughness increases, enhancing the SHP behavior of the coating. Similarly, the Ni 2p spectrum exhibits a set of characteristic peaks (Figs. 5e and 6f) with main doublets located around 852–856 eV (Ni 2p₃/₂) and 870–873 eV (Ni 2p₁/₂), corresponding to Ni²⁺ species such as NiO and Ni(OH)₂. The additional peaks at higher energies (≈ 861–883 eV) are satellite features, typically arising from multiplet splitting and shake-up processes associated with Ni²⁺ ions, further confirming the presence of oxidized nickel states on the surface^39^.

Overall, the increased intensities of C, Fe, and O signals along with the appearance of N species in the CQD-modified coating indicate greater surface functionalization and improved interfacial bonding, which contribute to enhanced roughness and so enhanced superhydrophobicity. These XPS findings are in good agreement with the EDX results, which also demonstrated an increase in Fe, C, and O content, the appearance of nitrogen, and a noticeable decrease in Ni intensity in the CQD-containing coating, confirming the consistency between both surface characterization techniques.

#### AFM and the roughness of the fabricated coatings

**Fig. 7:**
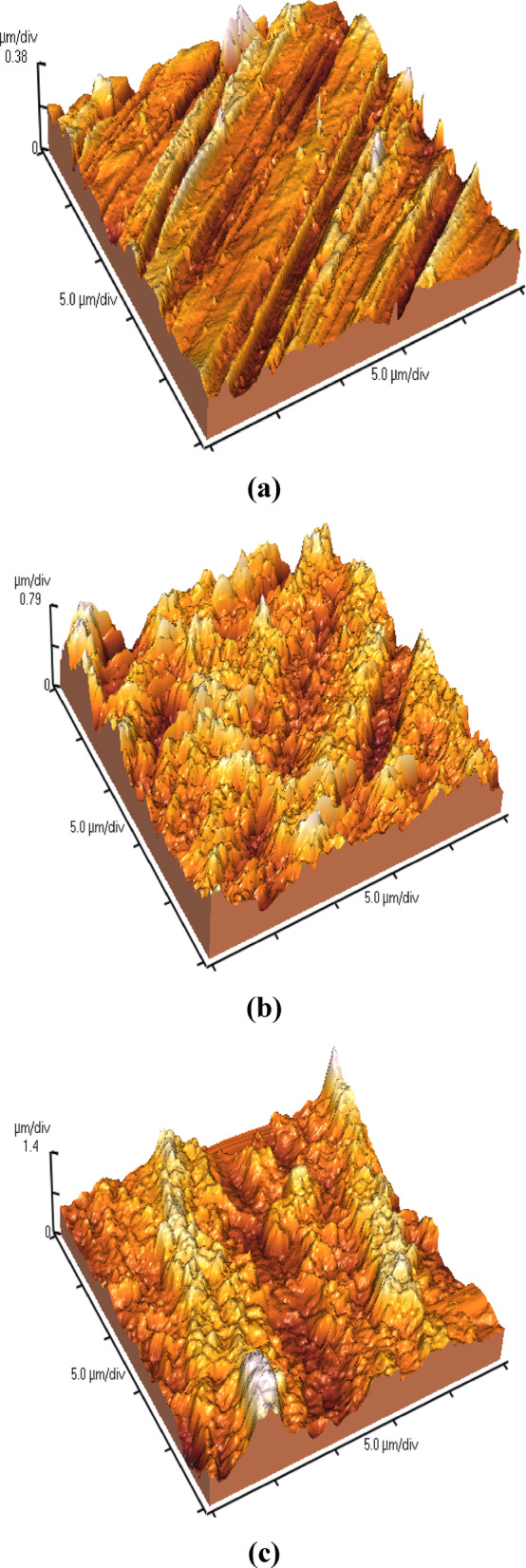
AFM 3D surface topographies of (**a**) bare steel substrate, (**b**) steel coated with SHP layer without CQDs, and (**c**) steel coated with SHP layer incorporating CQDs.

Atomic force microscopy (AFM) was used to inspect the roughness and surface morphology of the steel substrates under different conditions (Fig. 7). The uncoated steel surface (Fig. 7a) exhibited a relatively smooth morphology with an RMS roughness of 53.8 nm, indicating minimal topographical variations. After applying the SHP coating without CQDs (Fig. 7b), the roughness increased to 178.5 nm, reflecting the formation of micro/nanoscale hierarchical features essential for superhydrophobicity. With the incorporation of C-CQDs into the SHP coating (Fig. 7c), the roughness further increased significantly to 335.6 nm, accompanied by sharper and more irregular asperities. This progressive increase in roughness demonstrates the critical role of C-CQDs in enhancing hierarchical surface structures, thereby facilitating air entrapment and minimizing liquid–solid contact, which ultimately improves the SHP performance of the coating.

### Wettability results

**Fig. 8:**
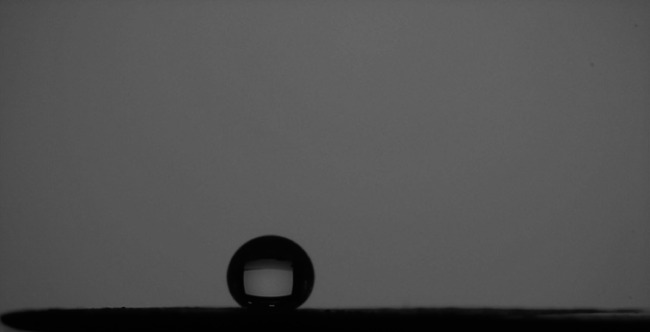
WCA image of the SHP coated steel surface without C-CQDs.

**Fig. 9:**
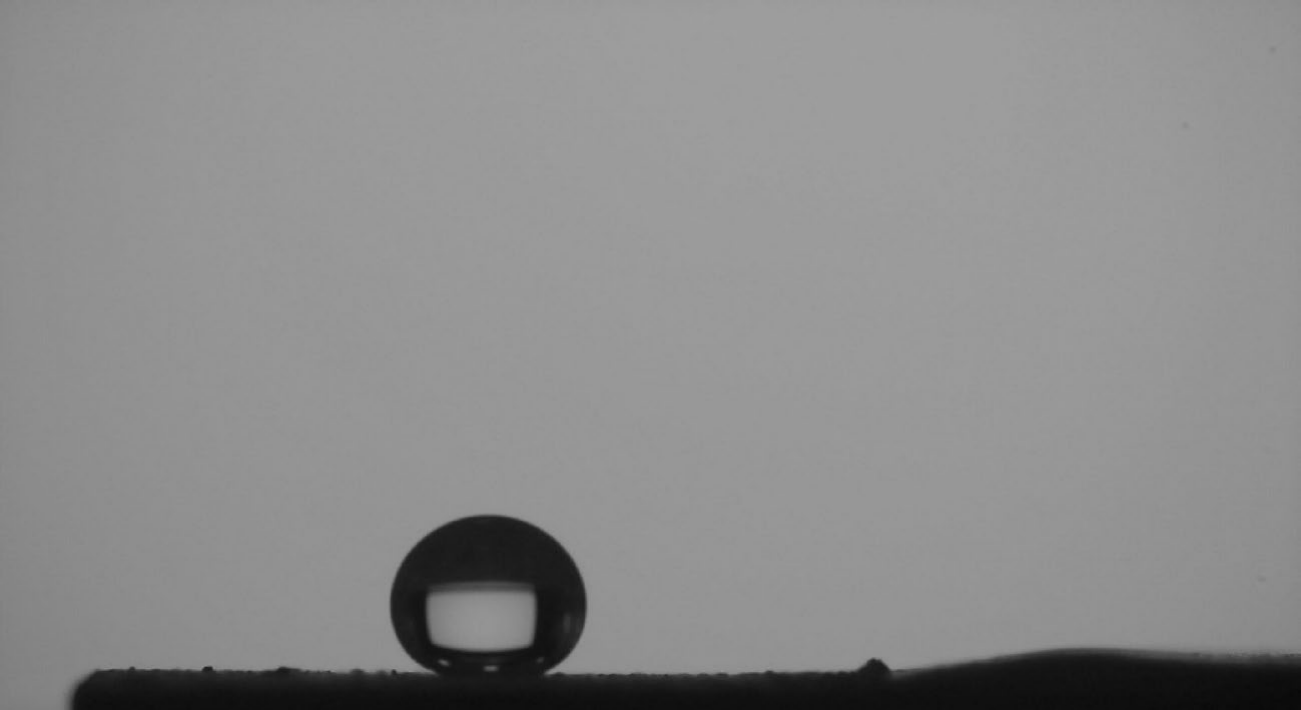
WCA image of the SHP-coated steel surface containing C-CQDs.

The wettability of the fabricated coatings was quantitatively assessed by measuring the WCA and WSA. As illustrated in Fig. 8, the SHP coating fabricated without C-CQDs exhibited a WCA of 156° ± 0.2 and a WSA of 3° ± 0.12, confirming the successful attainment of superhydrophobicity. A remarkable improvement was achieved with the incorporation of C-CQDs (Fig. 9), which yielded a WCA of 167°± 0.28 and an exceptionally low WSA of 1°± 0.1. Video S1 demonstrates the sliding of water droplets on the SHP coat incorporating CQDs. This superior performance demonstrates near-perfect water repellency and suggests excellent self-cleaning capability^40^.

This significant improvement in wettability is a direct consequence of the C-CQDs’ role in engineering the surface morphology. As established by the SEM and AFM analyses, the C-CQDs promote the formation of a denser and finer hierarchical micro/nanostructure. The uniform dispersion of C-CQDs and their surface functional groups critically enhances nanoscale roughness, which amplifies air entrapment and minimizes the liquid-solid contact area. The drastically reduced WSA of 1° is of particular importance. It confirms that the adhesive force between water droplets and the coating surface is minimal. This low adhesion ensures that water droplets roll off effortlessly with minimal tilt.

### Mechanical abrasion and chemical stability of the prepared SHP coated steel

**Fig. 10:**
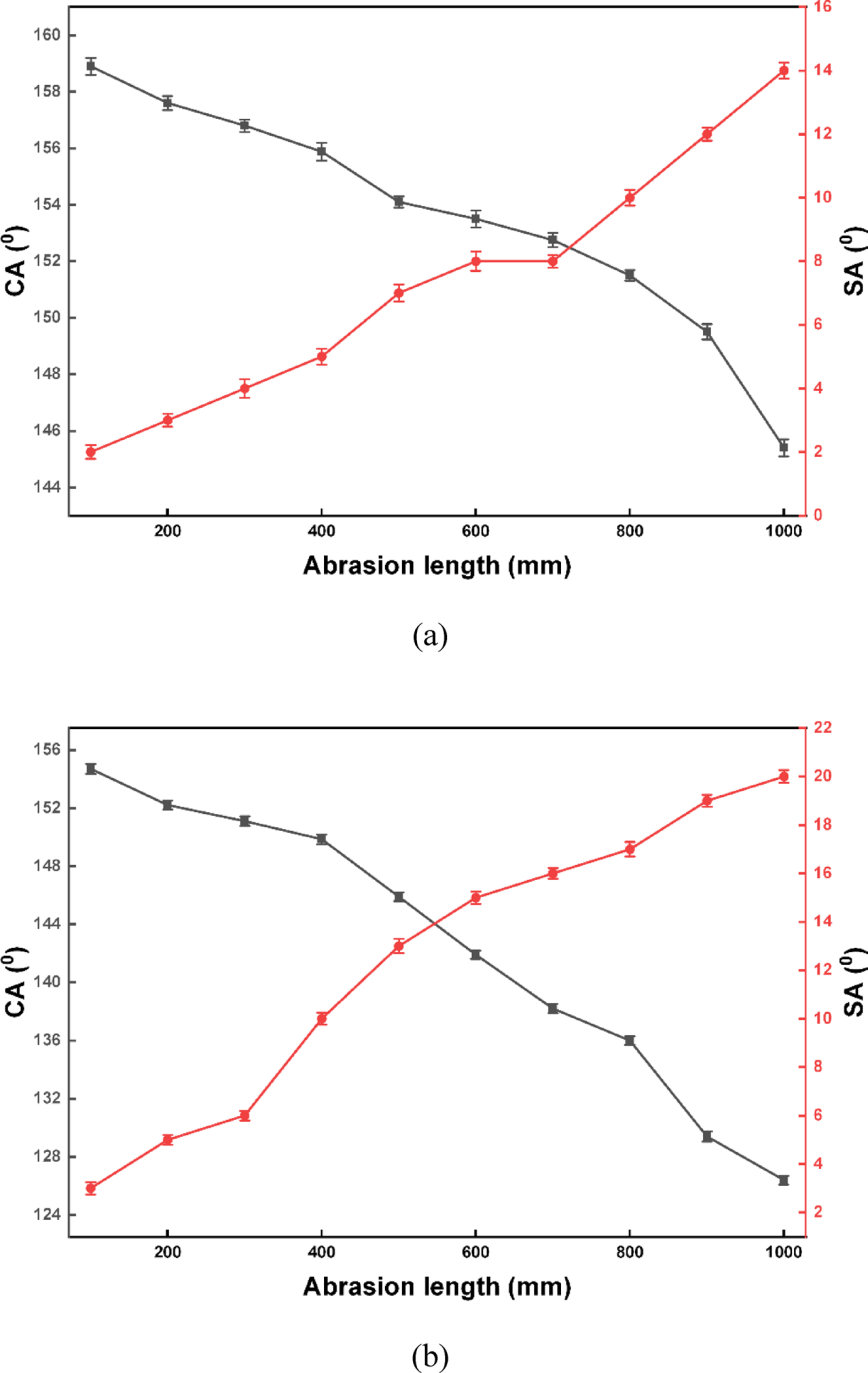
Mechanical abrasion resistance of the SHP coatings. Variation in WCA and WSA as a function of abrasion length for the coatings (**a**) with C-CQDs and (**b**) without C-CQDs.

The mechanical durability of the coatings was evaluated using a linear sandpaper abrasion test, in which the samples were abraded against 800-mesh sandpaper under a normal pressure of 3.0 kPa (Fig. 10). Under the present one-way abrasion protocol, the SHP coating incorporating C-CQDs maintained superhydrophobic behavior (WCA > 150° and WSA < 10°) up to an abrasion distance of 900 mm (Fig. 10a). In comparison, the coating without C-CQDs lost superhydrophobicity after approximately 400 mm, as the WCA decreased below 150° and the WSA exceeded 10° (Fig. 10b). These results conclusively show that the addition of C-CQDs is crucial for enhancing the mechanical abrasion resistance of the SHP coating. The data are presented as mean ± SD from repeated measurements, and error bars are included in Fig. 10^41^.

Because abrasion durability is strongly dependent on the test protocol (applied load/pressure, sandpaper grit, stroke definition, and one-way vs. reciprocating motion), direct comparison across studies should be made with caution. Nevertheless, representative literature reports indicate that many superhydrophobic coatings lose their performance after relatively short abrasion distances under common test conditions. For example, abrasion distances on the order of ~ 600 mm have been reported for electrodeposited superhydrophobic coatings (e.g., Mg(OH)_2_-based systems and template-free electrodeposited coatings on steel)^42,43^, whereas shorter abrasion distances of ~ 200–220 mm have also been reported for other coatings (e.g., copper plating modified with 1-octadecanethiol and stearic acid–TiO₂/Zn composite coatings) under their respective test condition^44,45^.

**Fig. 11:**
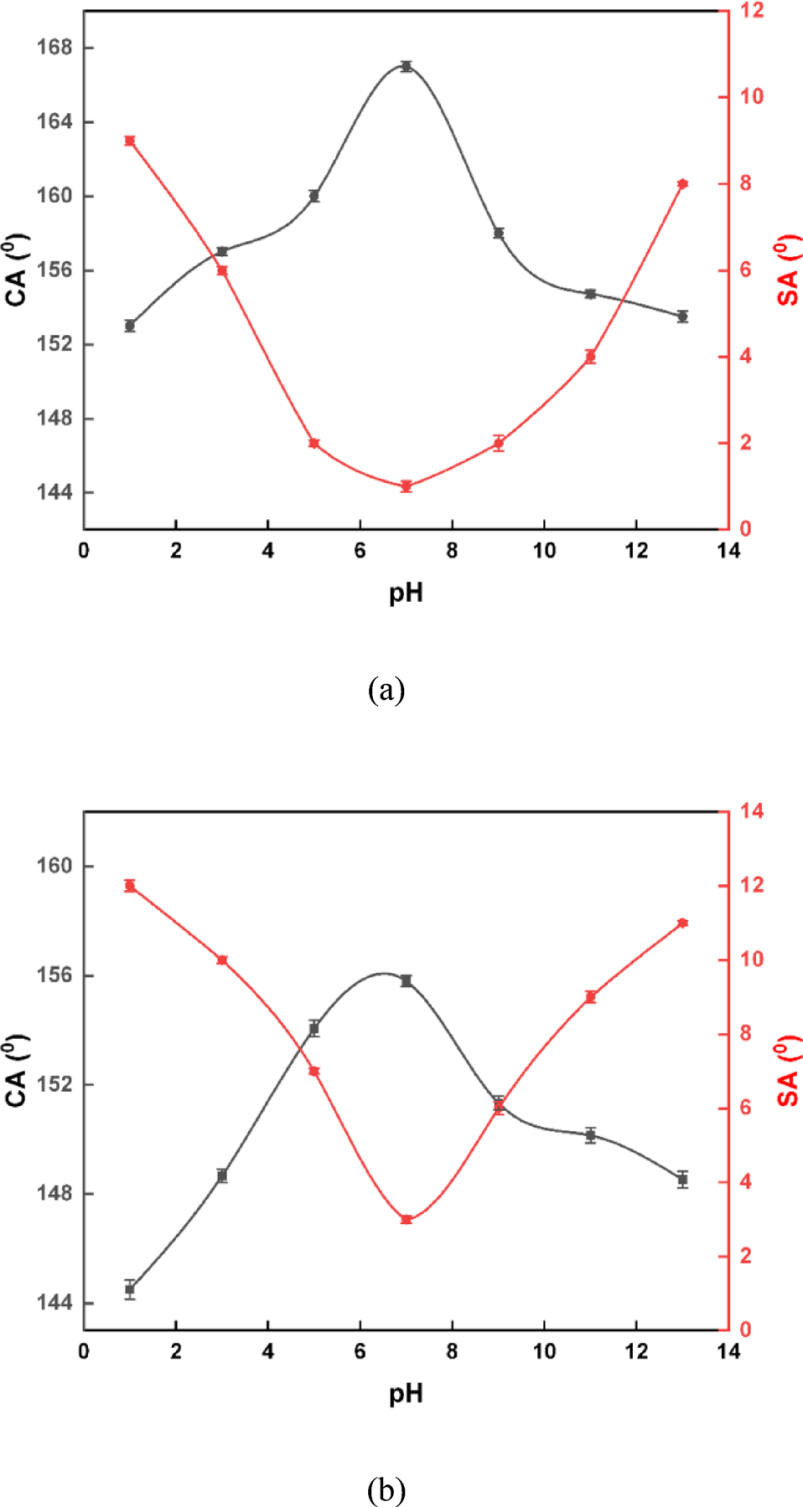
WCA and WSA measurements of SHP coatings on steel substrates as a function of pH (1–13): (**a**) SHP coating incorporating C-CQDs and (**b**) SHP coating without C-CQDs.

The chemical stability of the prepared SHP coatings was rigorously evaluated by immersing them in aqueous solutions with a wide pH range, from 1 to 13. The results (Fig. 11) indicate that the incorporation of C-CQDs significantly enhanced the coating’s robustness and hydrophobic performance. For the SHP coating with C-CQDs (Fig. 11a), the WCA remained impressively high, consistently above 153°, and the WSA stayed below 9° across the entire pH spectrum. The optimal performance was observed at a neutral pH of 7, with a maximum WCA of 167° ± 0.2 and a minimum WSA of just 1°± 0.1. In contrast, the coating without C-CQDs (Fig. 11b) failed to preserve its superhydrophobicity under harsh acidic and alkaline conditions. It only met the superhydrophobicity criteria within a narrower pH range of 5 to 9, with its peak performance at pH 7 (WCA=156°± 0.2, WSA=3°± 0.12), while at other pH values, it fell short of the required threshold. The data clearly show that the C-CQD-enhanced coating not only possesses superior super-hydrophobicity but also exhibits remarkable stability in both highly acidic and alkaline environments, confirming its potential for reliable performance in diverse applications. The data are reported as mean ± SD, and error bars are included in Fig. 11^46^. The enhancement in durability produced by C-CQDs can be attributed to both morphological and interfacial effects. First, the incorporation of C-CQDs during electrodeposition increases nucleation density and promotes grain refinement, yielding a more compact and uniformly distributed hierarchical texture rather than loosely packed asperities. Such a denser architecture is less prone to mechanical removal and delays the loss of the Cassie–Baxter state during abrasion. Second, the C-CQDs possess abundant oxygen- and nitrogen-containing surface groups (FTIR/XPS), which can strengthen interfacial interactions within the deposited layer and with the substrate, improving cohesion/adhesion and reducing defect density. Together, these effects reduce connected pore pathways for electrolyte penetration, supporting the improved stability under harsh pH and the higher corrosion resistance observed for the C-CQD-containing coating.

### Corrosion resistance of the prepared SHP coated steel

#### Electrochemical impedance spectroscopy results

**Fig. 12:**
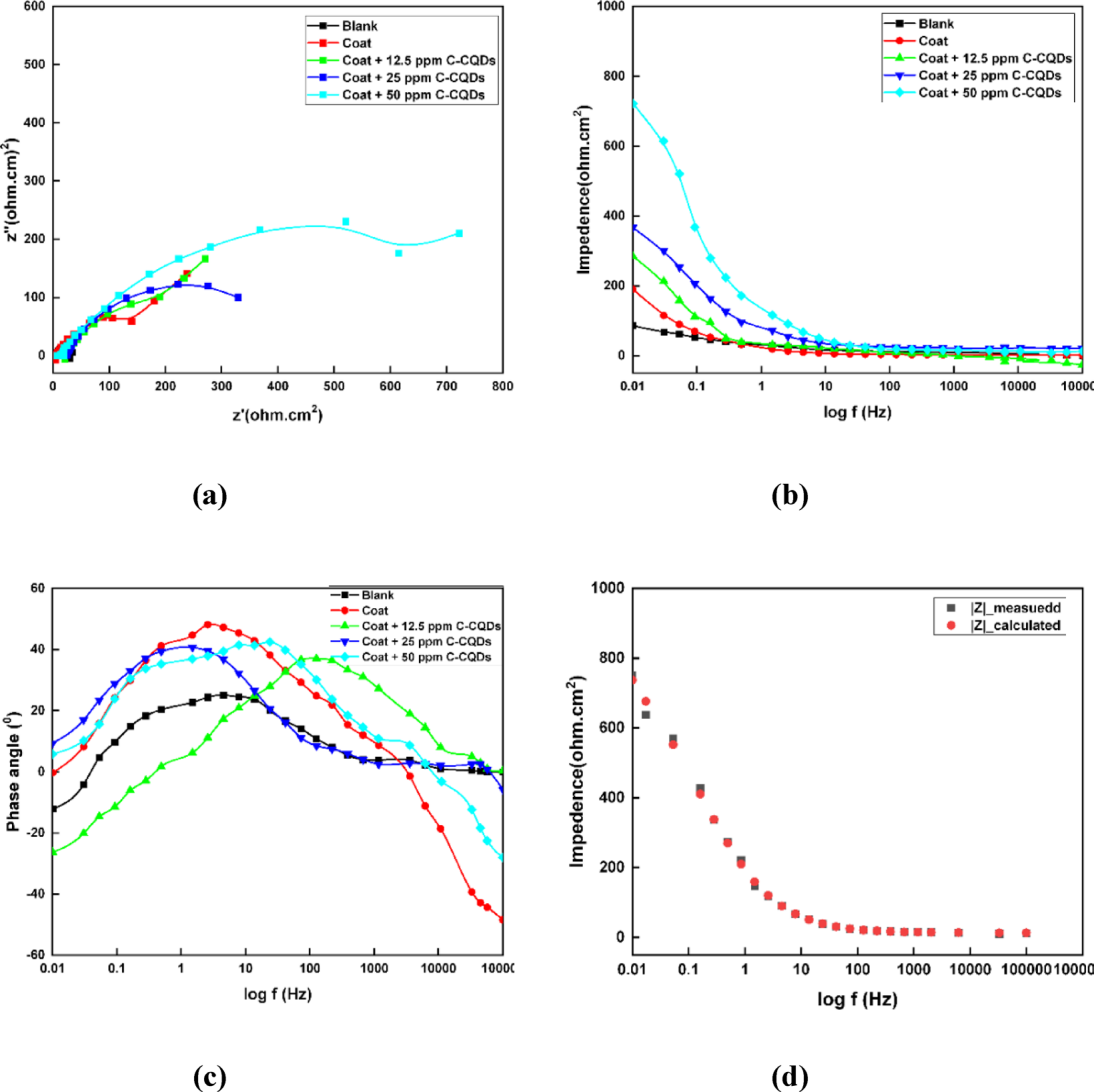
(**a**) Nyquist, (**b**) Bode, (**c**) phase-angle, and (**d**) the corresponding fitting curve plots for uncoated carbon steel (blank), SHP-coated steel without C-CQDs, and coated steel with different concentrations of C-CQDs after immersion in 0.5 M NaCl solution.

Figure 12 displays the Nyquist, Bode, and phase angle plots illustrating the electrochemical response of uncoated carbon steel, SHP-coated steel without C-CQDs, and that with C-CQDs immersed in a 0.5 M NaCl solution. As shown in the Nyquist diagram (Fig. 12a), a single depressed semicircle is observed for all samples, characteristic of a charge transfer–controlled corrosion process. The semicircle diameter, which is due to the charge transfer resistance (Rct), increases markedly after coating and attains its highest value for the CQD-modified coating, signifying enhanced corrosion resistance. This improvement reflects the role of C-CQDs in promoting a more uniform and compact coating layer that effectively blocks ionic diffusion and electron transfer.

The Bode plots (Fig. 12b) further support this behavior, where impedance values at low frequencies significantly increase upon coating, and the maximum values are achieved for the coating containing CQDs. This observation indicates the development of a more resistive barrier layer at the metal–electrolyte interface, reducing the corrosion rate. The phase angle plot (Fig. 12c) provides critical insight into the corrosion protection mechanism. The presence of a phase angle approaching − 45° in the low-frequency region is a definitive characteristic of a Warburg impedance (W). This confirms that the corrosion process is no longer primarily dominated by the charge transfer resistance at the interface but is instead dominated by the corrosive species diffusion (e.g., Cl^−^, O₂, H⁺) through a protective layer. Two relaxation time constants are apparent. The high-frequency time constant is due to the SHP coating. The low-frequency time constant is attributed to the steel interface.

**Fig. 13:**
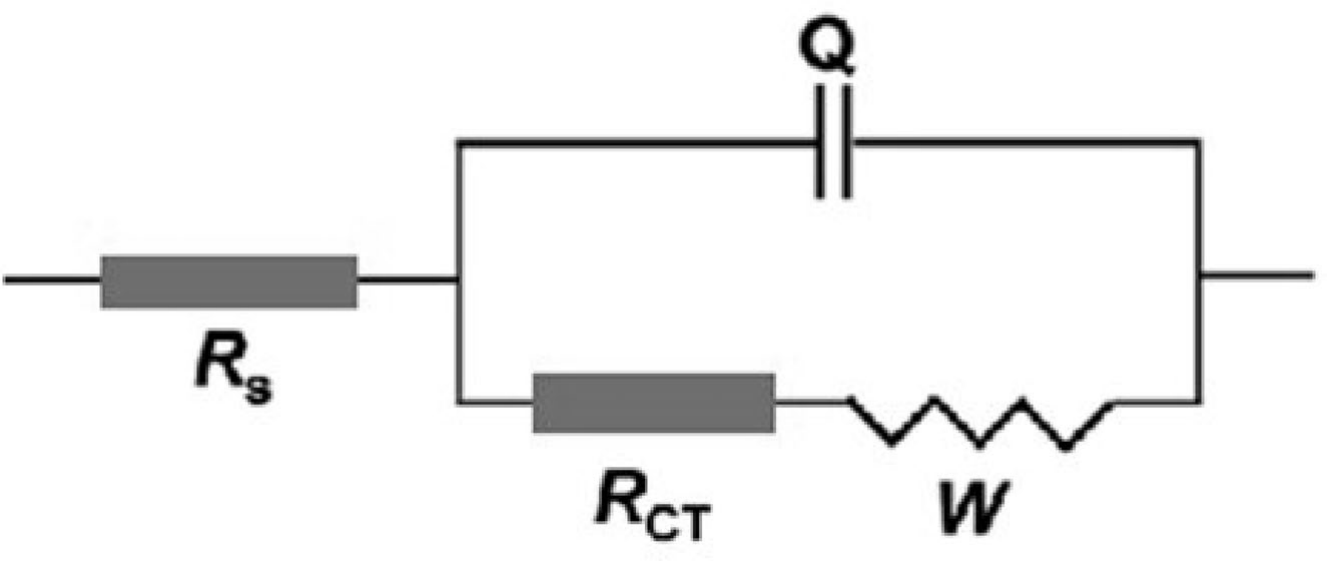
Equivalent electrical circuit model used for fitting the EIS data.

The experimental data of the EIS technique were fitted via the equivalent circuit shown in Fig. 13, which includes constant phase element (Qdl), solution resistance (R_s_), charge transfer resistance (R_ct_), and Warburg diffusion (W). The good agreement between experimental and fitted data (Fig. 12d) validates the accuracy of the proposed model. All parameters and measurement data are highly accurate, with errors of less than 3%. The inhibition efficiency was measured from Eq. (1)^47^.

1 $$\% {\mathrm{P}}={\text{ }}\left[ {\left( {{{\mathrm{R}}_{{\mathrm{ct}}}}-{\text{ R}}_{{{\mathrm{ct}}}}^{{\mathrm{o}}}} \right)/{{\mathrm{R}}_{{\mathrm{ct}}}}} \right]{\text{ }} \times {\mathrm{1}}00$$

Considering that R_ct_ represents the charge transfer resistance for SHP-coated steel with and without C-CQDs, while R°_ct_ represents the charge transfer resistance for uncoated steel.

The electrochemical parameters attained from the fitting are summarized in Table 2. A slight variation in Rs indicates a negligible change in solution conductivity, while R_ct_ exhibits a substantial increase from 42.9 Ω·cm² for the uncoated steel to 702.2 Ω·cm² for the C-CQD-coated surface, confirming superior corrosion inhibition. Concurrently, the double-layer capacitance (C_dl_) decreases from 5.2 × 10⁻³ to 1.4 × 10⁻³ µF·cm⁻², suggesting a reduction in the active surface area exposed to the electrolyte and the formation of a thicker, more compact SHP film. The inhibition efficiency (η%) also increased from 78.5% for the unmodified coating to 93.9% for the C-CQD composite, demonstrating the synergistic protective effect of CQDs through enhanced coating uniformity and surface passivation.

**Table 2:** Electrochemical impedance spectroscopy (EIS) parameters for uncoated carbon steel, SHP-coated steel without and with different concentrations of C-CQDs in 0.5 M NaCl solution.

Substrate	*R*_s_ ohm.cm^2^	*R*_ct_ ohm.cm^2^	Q_dl_ µF.cm^− 2^	*n*	C_dl_	% η
Bare steel	9.2	42.9	0.0086	0.55	5.2 × 10^− 3^	--
Coat	6.5	200.1	0.0026	0.78	2.6 × 10^− 3^	78.5
Coat + 12.5 ppm C-CQDs	16.3	345.8	0.0035	0.61	3.9 × 10^− 3^	87.6
Coat + 25 ppm C-CQDs	18.2	425.5	0.0053	0.67	7.9 × 10^− 3^	89.9
Coat + 50 ppm C-CQDs	12.9	702.2	0.0014	0.62	1.4 × 10^− 3^	93.9

#### Potentiodynamic polarization results

**Fig. 14:**
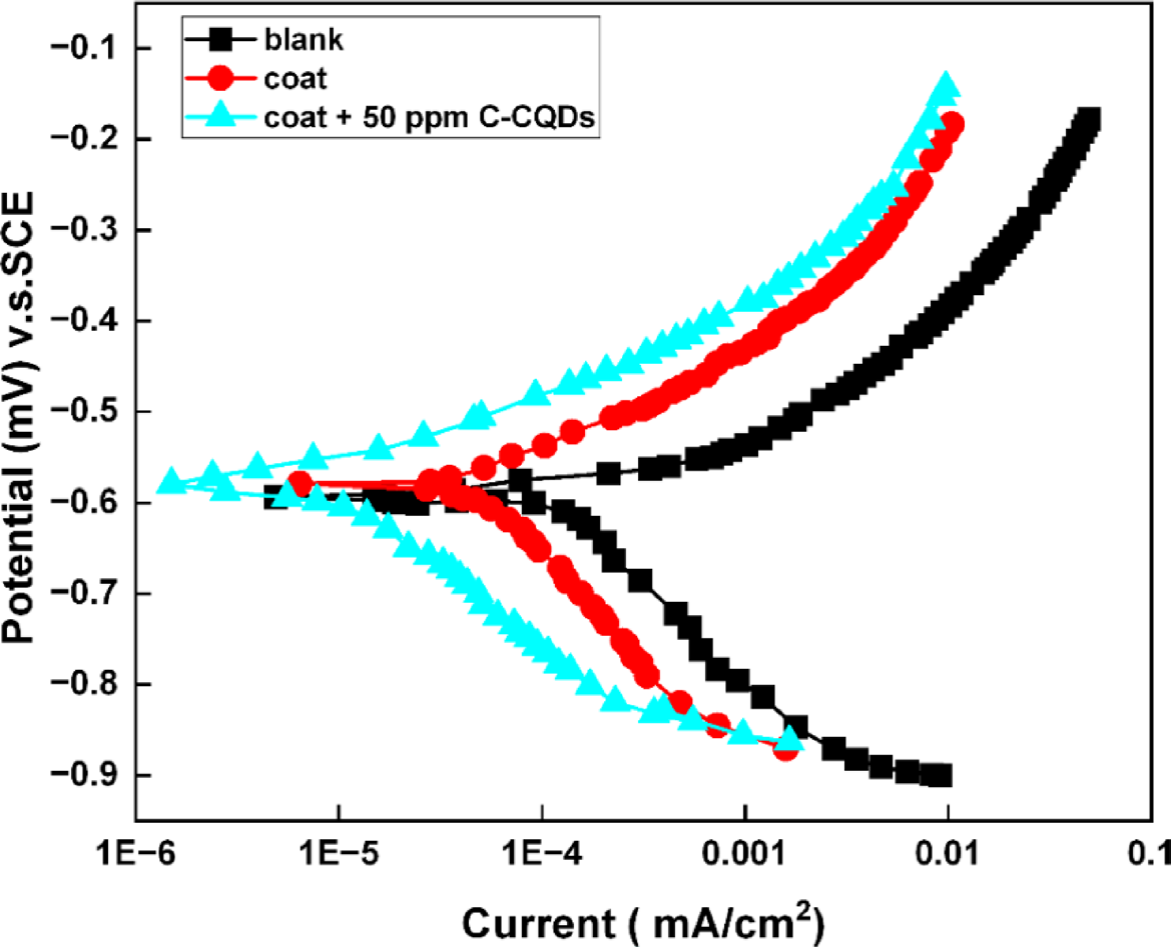
Potentiodynamic polarization curves of bare steel, SHP-coated steel, and coated steel with C-CQDs in 0.5 M NaCl solution.

The potentiodynamic polarization curves (Fig. 14) illustrate the corrosion performance of uncoated carbon steel and the SHP coatings without and with the optimum concentration of C-CQDs (50 ppm) in 0.5 M NaCl solution. For the SHP-coated steel, a shift occurs of both anodic and cathodic branches, demonstrating that the coating functions as an excellent mixed-type inhibitor. It does not merely block one half of the corrosion reaction but provides a comprehensive barrier that retards both the dissolution of the metal and the reduction of the corrosive agent. This leads to a profound decrease in the overall corrosion current density, which quantitatively translates to a much lower corrosion rate and superior protection performance for the coated steel.

The electrochemical parameters gotten from Tafel extrapolation are summarized in Table 3, and the inhibition efficiency (${\%}{\upeta\:})\:$was determined using Eq. (2)^48^:

2 $$\%{\upeta\:} =[ ( \: {\mathrm{i}}_{\mathrm{c}\mathrm{o}\mathrm{r}\mathrm{r}}^{\mathrm{o}}\: - \:{\mathrm{i}}_{\mathrm{c}\mathrm{o}\mathrm{r}\mathrm{r}}) / {\mathrm{i}}_{\mathrm{c}\mathrm{o}\mathrm{r}\mathrm{r}}^{\mathrm{o}}\: ] \times 100$$

where $\:{\mathrm{i}}_{\mathrm{c}\mathrm{o}\mathrm{r}\mathrm{r}}^{\mathrm{o}}\:$and $\:{\mathrm{i}}_{\mathrm{c}\mathrm{o}\mathrm{r}\mathrm{r}}$represent the corrosion current densities of uncoated and coated samples, respectively.

The corrosion current density quantitatively demonstrates the coating’s enhanced protection. The uncoated steel exhibited an i_corr_ of 0.3063 mA·cm⁻². This value decreased to 0.0704 mA·cm⁻² with the SHP coating without C-CQDs and dropped dramatically to 0.0211 mA·cm⁻² for the coating with 50 ppm C-CQDs. This decrease in the i_corr_ with the addition of C-CQDs confirms the critical role of C-CQDs in forming a superior barrier that effectively suppresses charge transfer and corrosion reactions.

The corrosion potential (E_corr_) of the SHP coatings shifted slightly toward more positive values compared to the blank sample, signifying a reduction in the corrosion tendency of the steel. The anodic and cathodic Tafel slopes (βa and βc) also increased in the presence of the coatings, indicating that the films act as physical barriers that hinder both anodic and cathodic processes. The polarization resistance and inhibition efficiency improved consistently, with the latter reaching 93% for the C-CQDs-modified coating. This improvement is due to the synergistic effect of the CQDs, which promote the formation of a denser and more uniform SHP layer, minimizing active sites for electrolyte penetration and effectively isolating the metal surface from the corrosive environment. These results are in excellent agreement with the EIS results, confirming the superior anti-corrosive performance of the C-CQD-enhanced SHP coating.

**Table 3:** Electrochemical polarization parameters for bare steel, SHP-coated steel without and with C-CQDs in 0.5 M NaCl solution.

Substrate	- E_corr_ (mV)	i_corr_ (mA cm^− 2^)	β_a_	-β_C_	% η
			mV/decade mV/decade		
Bare steel	588	0.3063	77.9	339.8	–
Coat	564	0.0704	79.2	399.1	77.0
Coat + C-CQDs	560	0.0211	68.7	391.7	93.1

#### Mechanism of corrosion protection

**Fig. 15:**
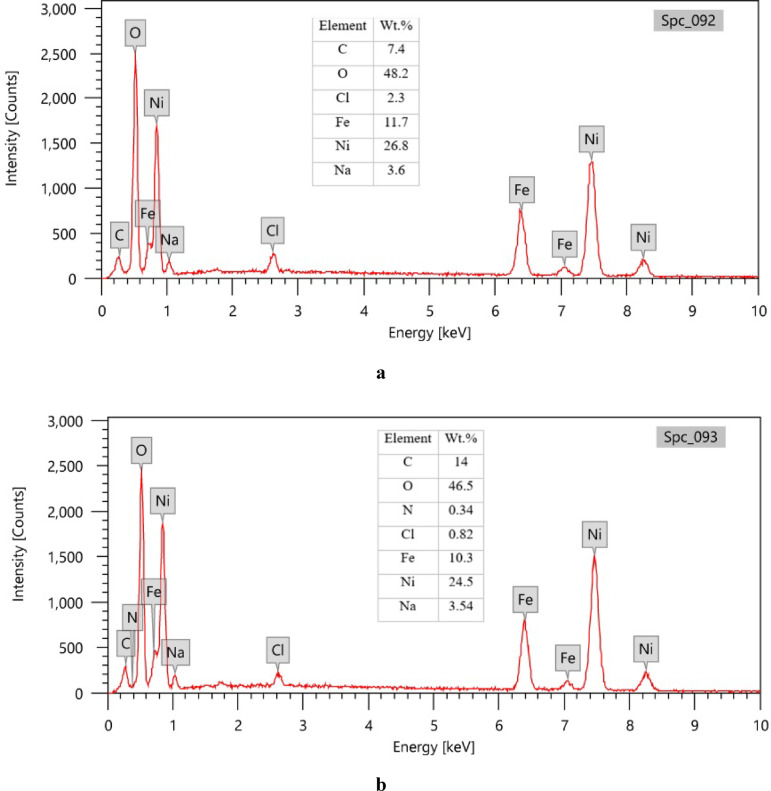
EDX spectra of (**a**) SHP-coated steel without C-CQDs and (**b**) SHP-coated steel incorporating 50 ppm C-CQDs after immersion in 0.5 M NaCl for 24 h.

Bare steel readily adsorbs water molecules on its surface and is therefore susceptible to chloride-induced corrosion. In chloride-containing environments, Cl⁻ can accumulate on the steel surface and promote corrosion reactions through the formation of intermediate species such as [FeClOH]⁻^49^. In contrast, when steel is protected by SHP coating, the micro-/nanostructures are covered with a low-surface-energy material, and the voids between surface asperities can be filled with trapped air. This trapped air layer acts as an effective barrier between the substrate and the corrosive electrolyte, reducing the real liquid–solid contact area and hindering the transport of aggressive ions (e.g., Cl⁻) toward the underlying surface. As shown in Fig. 15, the EDX spectra after immersion confirm the presence of oxygen-containing species on the surface, consistent with the formation of oxygen-rich corrosion products such as iron hydroxides/oxides (e.g., Fe(OH)_3_ and Fe_2_O_3_). Comparing Fig. 15a and b, the CQDs-containing coating shows a lower chlorine signal after immersion, indicating reduced chloride accumulation/ingress. Moreover, SHP surfaces in neutral solutions may exhibit a net negative interfacial charge because the isoelectric point of many SHP materials lies in the acidic range (approximately pH 2–4)^50^. A negatively charged interface can reduce the local concentration of chloride anions near the surface, thereby improving corrosion resistance. In the present work, C-CQDs contain abundant electronegative functional groups (e.g., oxygen- and nitrogen-containing groups), which may influence interfacial interactions and ion distribution at the coating/electrolyte interface and discourage chloride accumulation. Consequently, the C-CQDs-assisted SHP coating is expected to lower the amount of Cl⁻ near the surface, in agreement with the comparison between Fig. 15a and b after immersion.

### Adsorption isotherm

**Fig. 16:**
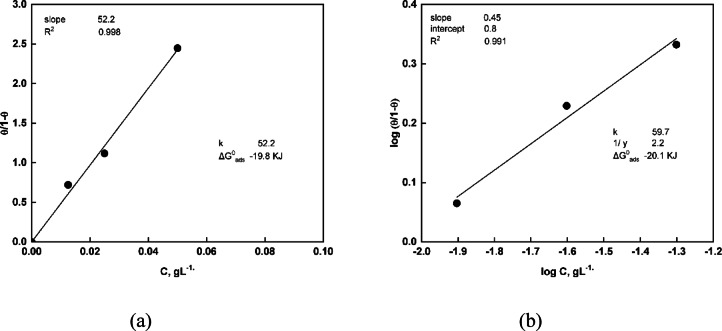
C-CQDs adsorption data on carbon steel in 0.5 M NaCl were fitted linearly using the impedance technique to (**a**) Langmuir; (**b**) Kinetic-Thermodynamic model.

Different adsorption isotherm models, including the Langmuir model, were applied to investigate and fit the adsorption behavior obtained from the EIS data. In addition, the kinetic–thermodynamic model was utilized to assess the mechanism of adsorption of C-CQDs on the carbon steel surface. The Langmuir adsorption isotherm (Fig. 16a) is represented by Eq. (3):

3 $$\:\begin{array}{cccc}&\:\frac{{\uptheta\:}}{1-{\uptheta\:}}={\mathrm{K}}_{\mathrm{ads}}\:{\mathrm{C}}_{\mathrm{CQD}}&\:&\:\end{array}$$

where $\:{\mathrm{C}}_{\mathrm{CQD}}$is the concentration of C-CQDs (mg/L), $\:{\mathrm{K}}_{\mathrm{ads}}$is the equilibrium adsorption constant, and $\:{\uptheta\:}\:$represents the surface coverage^51^.

The Kinetic–Thermodynamic model (Fig. 16b) is presented by Eq. (4):

4 $$\:\begin{array}{cccc}&\:\mathrm{log}\left(\frac{{\uptheta\:}}{1-{\uptheta\:}}\right)=\mathrm{log}{\mathrm{K}}^{{\prime\:}}+\mathrm{ylog}\mathrm{C}&\:&\:\end{array}$$

The corresponding binding constant $\:\mathrm{K}\:$is given by Eq. (5), where $\:\mathrm{y}\:$denotes the number of inhibitor molecules occupying a single active site, and $\:\frac{1}{\mathrm{y}}\:$signifies the number of active sites covered by one C-CQD molecule:

5 $$\:\begin{array}{cccc}&\:\mathrm{K}={{\mathrm{K}}^{{\prime\:}}}^{\left(\frac{1}{\mathrm{y}}\right)}&\:&\:\end{array}$$

To elucidate the adsorption mechanism of C-CQDs onto the steel substrate, the corrosion protection efficiency was calculated using EIS data. However, for the specific purpose of applying adsorption isotherm models, the protection efficiency (Table 4) is reported relative to the superhydrophobic coating without CQDs, rather than the bare steel. This approach isolates the incremental protective effect conferred solely by the adsorbed C-CQDs, allowing the equilibrium data between the C-CQDs concentration and this relative efficiency increase to be accurately fitted to various isotherm models (e.g., Langmuir or Kinetic–Thermodynamic model). The fit of the data to a specific model will then reveal whether the C-CQDs is chemically (chemisorption) or physically (physisorption) adsorbed to the steel surface.

**Table 4:** Protection efficiency values, relative to the unmodified SHP coating, used for the analysis of CQD adsorption isotherms.

Substrate	*R*_ct_ ohm.cm^2^	% η
Coat	200.1	–
Coat + 12.5 ppm C-CQDs	345.8	42.1
Coat + 25 ppm C-CQDs	425.5	53.0
Coat + 50 ppm C-CQDs	702.2	71.5

Figure 16 shows that both models exhibited strong linearity, confirming that the adsorption of C-CQDs on the steel surface in 0.5 M NaCl follows an ideal adsorption process. The Langmuir isotherm displayed a good correlation coefficient (R² = 0.9985), and the obtained high $\:{\mathrm{K}}_{\mathrm{ads}}\:$value reflects the strong affinity of C-CQDs toward the metal surface. From the Kinetic–Thermodynamic model, the occupancy factor $\:\frac{1}{\mathrm{y}}=2.2\:$indicates that each C-CQD molecule covers approximately 2.2 adsorption sites.

As shown in Fig. 16, and according to Eq. (6)^52^, the adsorption free energy values calculated from the models were comparable, with an average $\:{\Delta\:}{\mathrm{G}}_{\mathrm{ads}}^{\circ\:}=-20\:$kJ·mol^−1^:

6 $$\:\begin{array}{cccc}&\:{\Delta\:}{\mathrm{G}}_{\mathrm{ads}}^{\circ\:}=-\mathrm{R}\mathrm{T}\mathrm{l}\mathrm{n}\left(55.5{\mathrm{K}}_{\mathrm{ads}}\right)&\:&\:\end{array}$$

Here, $$\:\mathrm{T}\ $$ is the absolute temperature, 55.5 denotes the molar concentration of water in the solution, and$\:\:\mathrm{R}\:$is the universal gas constant. Values of $\:{\Delta\:}{\mathrm{G}}_{\mathrm{ads}}^{\circ\:}$between − 20 and − 40 kJ·mol^−1^ indicate a mixed physicochemical adsorption mechanism. Accordingly, the adsorption of C-CQDs onto the carbon steel surface occurs through a combined physical and chemical interaction.

### Self-cleaning performance

To demonstrate the practical advantage of our material, the self-cleaning performance of the SHP coating enhanced with C-CQDs was evaluated. While standard SHP coatings are known to exhibit self-cleaning, the key difference lies in the efficiency and robustness of the process. As captured in supplementary video S2, water droplets rolled effortlessly and rapidly across the C-CQDs-coated surface, effectively picking up and removing contaminant particles without leaving any residue. The droplets exhibited minimal adhesion, leading to a swift and complete cleaning action that left the substrate perfectly clean and dry.

This excellent self-cleaning property is attributed to the synergistic effect of high surface roughness and low surface energy imparted by the C-CQDs. The C-CQDs’ nanoscale features promote the Cassie–Baxter wetting state, where air pockets trapped beneath the water droplets minimize solid–liquid contact, enabling the droplets to easily roll off and carry away contaminants. These findings confirm that incorporating C-CQDs not only enhances surface hydrophobicity but also significantly improves the functional stability of the coating for contamination resistance and corrosion protection.

In addition to corrosion protection, the present coating may offer potential anti-fouling/antibacterial benefits. Conocarpus-derived materials have been reported to exhibit antibacterial activity^53^, suggesting that the biomass origin of the C-CQDs could contribute to antimicrobial-relevant surface chemistry. Moreover, the superhydrophobic state can reduce bacterial adhesion and biofouling by minimizing the effective liquid–solid contact area and shortening the residence time of aqueous droplets on the surface. Therefore, the combination of a low-adhesion superhydrophobic interface and the presence of C-CQDs may be advantageous for applications where biofouling mitigation is desired. A systematic antibacterial evaluation (e.g., bacterial adhesion and viability assays) will be considered in future work.

## Conclusion

This study successfully demonstrates the following key findings:

- Sustainable Fabrication: A durable, eco-friendly superhydrophobic (SHP) coating was fabricated on steel using carbon quantum dots derived from Conocarpus lancifolius (C-CQDs) as a multifunctional additive.
- Enhanced Surface Morphology: The C-CQDs acted as nucleation agents during electrodeposition, promoting the formation of a denser, finer hierarchical structure with superior nanoscale roughness. This resulted in exceptional superhydrophobicity (WCA = 167°, WSA = 1°) and self-cleaning performance.
- Confirmed Surface Modification: XPS and EDX analyses verified successful surface functionalization, indicated by increased C, Fe, and O signals and the introduction of N species. This confirms improved interfacial bonding and directly contributes to the enhanced surface roughness and hydrophobicity.
- Stability and Corrosion Resistance: The C-CQD-modified coating exhibited remarkable resilience, maintaining superhydrophobicity (WCA > 153°) after extensive abrasion (900 mm) and exposure to a wide pH range (1–13). It achieved a high corrosion protection efficiency of 93.1% (compared to 78.5% for the unmodified SHP coating) by forming a compact barrier that significantly reduced corrosion current density.
- Adsorption Mechanism: The adsorption of C-CQDs onto the steel surface was successfully modeled. The data fit both the Langmuir isotherm and a kinetic-thermodynamic model. The calculated standard adsorption free energy (ΔG°= −20 kJ mol^−1^) indicates a spontaneous mixed physico-chemical adsorption mechanism.

## Supplementary Information

Below is the link to the electronic supplementary material.

Supplementary Material 1

Supplementary Material 2

Supplementary Material 3

## Data Availability

The data that support the findings of this study are available from the corresponding author upon reasonable request.
