# Supplementary material for: Biomass-derived carbon quantum dots for the fabrication of a durable, self-cleaning, and corrosion-resistant superhydrophobic coating on steel
Source: Sci Rep. 2026 Apr 30;16:13897. doi: 10.1038/s41598-026-47261-8 (PMC13133399; doi:10.1038/s41598-026-47261-8)
Supplement: Supplementary file 1 — Supplementary Material 1 [file 41598_2026_47261_MOESM1_ESM.docx]

**Biomass-Derived Carbon Quantum Dots for the Fabrication of a Durable, Self-Cleaning, and Corrosion-Resistant Superhydrophobic Coating on Steel**

M. E. Mohamed^a,b^, B.A. Abd-El-Nabey ^a^, A. Ezzat^a*^

^a^ Chemistry Department, Faculty of Science, Alexandria University, Egypt.

^b^  Faculty of Advanced Basic Sciences, Alamein International University, New Alamein City, Matrouh Governorate, Egypt.

*E-mail: [alaaezzatmohamed@gmail.com](mailto:alaaezzatmohamed@gmail.com) (A.E)

https://drive.google.com/file/d/1H1IdHuibww1nAbK1C6l_8M_it5kmpV41/view?usp=drive_link

**Video S1:** The sliding of water droplets on the SHP coat incorporating CQDs.

https://drive.google.com/file/d/1HzsFvYNJHBxpCIn7dXdENUYwnL7uS-db/view?usp=drive_link

**Video S1:** The sliding of water droplets across the C-CQD-coated surface containing a contaminant.
